# Locked Down: Experiences of Domestic Violence in Central India

**DOI:** 10.9745/GHSP-D-21-00630

**Published:** 2022-08-30

**Authors:** Anusha Kamath, Anita Yadav, Jyoti Baghel, Shuchita Mundle

**Affiliations:** a Department of Obstetrics and Gynecology, All India Institute of Medical Sciences, Nagpur, India.

## Abstract

**Introduction:**

The National Family Health Survey (NFHS-4) data show that 31.1% of women faced domestic violence in 2015–2016, with no reduction since 2005–2006. Emerging data show that since the outbreak of the coronavirus disease (COVID-19), reports of intimate partner violence have increased worldwide because of mandatory lockdowns to curb the spread of the virus. The nature of the current pandemic, which has forced women to spend time with their abusers and restricted their mobility, has made them more vulnerable to abuse. We aimed to determine the proportion of domestic violence among Indian women during the lockdown period.

**Methods:**

This is a cross-sectional study, conducted in June–August 2020 on married, Indian women of reproductive age who attended the obstetrics-gynecology outpatient department during the COVID-19 pandemic. The Abuse Assessment Screening questionnaire validated during NFHS-4 (2015–2016) was used as a data collection tool. Our primary objective was to determine the proportion of women presenting to the hospital who were exposed to domestic violence during the lockdown period. Analysis was done using SPSS Statistics V22.0.

**Results:**

A total of 412 women were recruited for the study. The mean age was 23 years (range 19–52 years). The prevalence of domestic violence in any form was found to be 32.5%, with the majority being verbal abuse in the form of insults, threats of physical violence, or public humiliation.

**Conclusion:**

The results of this study indicated a high proportion of women with a history of domestic violence during the pandemic. Thus, health care policy makers and experts involved in women’s care—particularly during crisis conditions such as the COVID-19 pandemic—should be aware of the extent of this problem and take measures to properly address it.

## Abstract (precis)

Mandatory lockdowns to reduce the spread of COVID-19 have led to increased reports of domestic violence experienced by women globally. Because health care workers focus on the pandemic response, women who experience domestic violence may not seek help and may remain a neglected population.

## Teaser

**Key Findings:**

- Among our sample of women, we found a prevalence of domestic violence (32.5%), with the most common form being verbal abuse and the majority of abuse committed by the spouse or mother-in-law.
- Only 6.8% of these women reported calling helplines or lodged a formal complaint.
- While the medical community focuses on controlling the spread of coronavirus disease (COVID-19), women who experience domestic violence may be neglected in light of health care system challenges caused by the pandemic.

**Key Implications:**

- Health care workers should learn the signs of domestic violence and understand the role that lockdowns play in increasing domestic violence rates.
- Policy makers should integrate domestic violence and mental health care into overall public health planning as well as emergency responses, understanding that multiple sectors must coordinate to ensure comprehensive support for women.

## INTRODUCTION

In India, domestic violence is defined by Parliament in the Protection of Women from Domestic Violence Act 2005 as “physical, sexual, verbal, emotional, and economic abuse against women by a partner or family member residing in a joint family.”^1^ The 2015–2016 India National Family Health Survey (NFHS-4) data showed that 31.1% of women aged 15–49 years faced physical and sexual domestic violence; most commonly perpetrated by the current husband.^2^ It also reported that 4% of women reported having experienced domestic violence during their pregnancies.^2^

Worldwide, 30% of women experience physical or sexual violence by their partners throughout their lives.^4^^,^^5^ The United Nations has acknowledged domestic violence as a violation of basic human rights. Victims of domestic violence are abused inside their own homes, which is expected to be a secure environment, usually by the people they trust the most.^4^ Violence manifests in different forms including coercive control, physical abuse, emotional abuse, or sexual violence; unfortunately, usually only physical violence receives attention. Violence against women can result in injuries and serious physical, mental, sexual, and reproductive health problems, including sexually transmitted infections, HIV, and unplanned pregnancies.^4^

Despite the protections promised by India’s Domestic Violence Act of 2005, women often hesitate to seek legal protection and tend to informally confide in friends and family members. This could be due to a lack of knowledge, mistrust of the police and justice systems, or poor access to legal help.^3^

Emerging data show that since the outbreak of coronavirus disease (COVID-19), mandatory lockdowns to curb the spread of the virus,^6^^,^^7^ have led to a “horrifying global surge in domestic violence” according to United Nations Secretary-General António Guterres^8^ and “a shadow pandemic of violence against women and girls.^9^”

Global reports of domestic violence have increased during the COVID-19 pandemic, as lockdowns keep women isolated with their abusers.

Violence in home situations has likely increased for several reasons: living with families in close confined spaces during lockdowns can lead to conflicts, as people are denied other ways of overcoming stress or boredom. Financial insecurity has caused livelihood issues such as job losses, prolonged unemployment, reduced income, debts, and food insecurity.^3^^,^^10^ This can also lead to chronic stress, which is well known to play a significant role in causing poor mental health and psychiatric disorders.^1^^,^^11^

The nature of lockdowns during the current pandemic, which has forced women to spend more time with their abusers and restricted their mobility, has made them more vulnerable to abuse.^12^ Women may also avoid seeking health care for injuries caused by abuse out of fear of possible COVID-19 infection. Additionally, during a pandemic, health professionals may have several other preoccupations, such as resource scarcity, lack of personal protection, lack of a private environment for consultation, and a focus on emergency medical care; therefore, they may fail to recognize the signs of possible violence.

We attempted to determine the proportion of women presenting to the hospital who were exposed to domestic violence and the factors associated with it among Indian women of reproductive age during the lockdown period in central India.

## METHODS

With the declaration of the pandemic, a strict lockdown was imposed in India, especially during the initial months (March–June 2020). The current cross-sectional study was conducted over 3 months between June 1–August 31, 2020, when the lockdown was relaxed to some extent. People had some freedom of movement, especially to attend health care facilities for reasons other than COVID-19.

The study was conducted at 2 health care facilities in Nagpur: the All India Institute of Medical Sciences, a tertiary care referral center and a teaching hospital, and the Urban Community Health Care Centre, Nandanwan, a secondary level health care unit.

For the study, we approached women availing services from the outpatient obstetrics/gynecology department in these 2 health care facilities. The inclusion criteria were women aged 18 years or older, currently married or in a relationship, and willing to provide written consent.

The situation during COVID-19 did not allow for the employment of new researchers for the study; hence, it was conducted by a team consisting of 2 senior residents and 4 nursing officers already working in the department. Besides an understanding of biomedical research, they were also specially trained for the study regarding informed consent procedures and the interview guide for data collection.

Researchers noted each participant’s sociodemographic profile, including age, education, employment status, and type of family. Jobs were classified as “unskilled” category if women were employed as laborers at construction sites, farm laborers, or domestic help (per the guidelines laid down by the Ministry of Labor and Employment).^13^ Jobs were considered “skilled” if women were involved in clerical work or employed as a beautician, receptionist, or teacher.^13^

We defined a nuclear family system as “a 2-generation family consisting of a father and mother and children or a single, possibly widow, parent and his/her children.” We defined joint or extended family as “3 or more generations lived together with both vertical and lateral extension having a single line of authority, either patrilineal or matrilineal.”^14^^,^^15^

Researchers took a brief reproductive history, including the presenting symptoms and menstrual and obstetric history. Then, researchers asked questions about the effect of the COVID-19 pandemic on the family environment, which enabled the interviewer to form a rapport with the client before moving on to personal questions about domestic violence. The questionnaire was used as an interview guide.

Participants’ experience with domestic violence was documented using the Abuse Assessment Screening questionnaire, which has been validated and used during NFHS-4 (2015–2016).^2^ The questionnaire explores the experience of violence by the woman in the family, emphasizing the physical, sexual, and verbal aspects of abuse (Box). A woman would be considered exposed to violence if she gave at least one positive response to each of the items related to physical, sexual, or emotional violence. They were required to comment on the frequency of the episodes during the previous 3 months: never, sometimes, often, or more. Women who chose “never” were those who had not experienced violence.

BOX **Categories of Responses to Abuse Assessment Questionnaire Used to Document Domestic Violence Among Women in Nagpur, India**

Women were considered to have experienced the following types of domestic violence if their responses to the abuse assessment questionnaire were affirmative.

Verbal domestic violence if the spouse ever:

- Publicly humiliated her
- Insulted her or made her feel bad
- Threatened her with harm

Physical domestic violence was categorized into less severe and severe types.

Less severe physical violence if the spouse ever:

- Pushed her, shook her, or threw something at her
- Slapped her
- Punched her with his fist or something harmful
- Kicked or dragged her

Severe physical violence if the spouse ever:

- Tried to strangle or burn her
- Threatened or attacked her with a knife, gun, or other weapon

Sexual violence if the spouse ever:

- Physically forced sexual intercourse when not wanted
- Forced other sexual acts when not wanted

The questionnaire also addressed their support-seeking preferences. During data collection, if researchers identified domestic violence, they offered women help in the form of referrals to counseling centers and helpline numbers.

Data were entered into a spreadsheet, with categorical data and continuous data recorded into numerical variables and expressed as mean and median and frequency, respectively. An unpaired t-test was performed to compare 2 group means. Chi-square and Fisher exact tests were done to determine the association between categorical variables. A *P* value of less than .05 was considered statistically significant. Data analysis was done using SPSS Statistics V22.0.

Before recruiting the participants, we informed women about the study’s goals and methods, their voluntary participation, confidentiality, privacy protection, and their right to quit the study at any stage of data collection. We recruited women who fulfilled the inclusion criteria after they gave their written informed consent. We assured the women of complete confidentiality and anonymity of the data. Because of the sensitive nature of the questions, privacy was of key priority throughout the face-to-face interview. The interview was conducted in a private room in the outpatient department (OPD) by a research team member.

### Ethics Approval

Approval for the study was obtained from the Institutional Ethical Committee, AIIMS Nagpur, (NoIEC/Pharmac/111/20, dated May 14, 2020).

## RESULTS

During the study period of 3 months, 587 women visited the obstetrics/gynecology department of the health facility. Of these, 41 (6.9%) were excluded (17 were girls aged younger than 18 years; 28 women were aged older than 18 years, but not currently married or in a relationship). An additional 130 (22.1%) women were not willing to participate in the study and were also excluded. Thus, after excluding the women who did not fit the study inclusion criteria or refused to give consent, 412 women were included in the study.

### Sociodemographic and Reproductive Profile

Baseline characteristics of the study participants (N=412) are listed in Table 1. Most of the women (97.1%) were literate; 39.5% of the women were graduates. It means they had some form of higher education after completing their schooling. Fifty percent of the women were housewives. Seventy percent of them resided in urban localities and nearly 60% had a nuclear family. Of their spouses, 96.3% were literate and a majority (45.3%) were self-employed. The spouses of 27.9% of respondents had a history of substance abuse, either in the form of chewing tobacco (n=51), smoking (n=66), or alcohol consumption (n=58).

**TABLE 1.** Sociodemographic Characteristics of Participants in a Study on Domestic Violence Among Women in 2 Health Care Facilities, Nagpur, India, June to August 2020

**Variables**	**No. (%), (N=412)**
Age, years	
19–29	195 (47.3)
30–39	149 (36.1)
40–49	48 (11.6)
≥50	20 (4.9)
Education	
Illiterate	12 (2.9)
Primary	12 (2.9)
Middle school	46 (11.1)
Secondary	62 (15)
Higher secondary	117 (28.3)
Graduate and above	163 (39.5)
Occupation	
Housewife	205 (49.7)
Unskilled worker	49 (11.8)
Skilled work	90 (21.8)
Self-employed	68 (16.6)
Education of husband	
Illiterate	16 (3.8)
Primary	12 (2.9)
Middle school	22 (5.3)
Secondary	61 (14.8)
Higher secondary	54 (13.2)
Graduate and above	247 (59.9)
Occupation of husband	
Unskilled worker	76 (18.4)
Office work	145 (35.2)
Self-employed	191 (46.3)
Residence	
Urban	289 (70.2)
Rural	123 (29.8)
Type of family	
Nuclear	284 (69)
Joint family with in-laws	114 (27.6)
Joint family with wife’s parents	6 (1.4
Extended joint family	1 (0.2)

Almost 50% of the women had been married for more than 5 years. Only 6.8% were married for less than 1 year. The reasons for attending the OPD were antenatal checkups (21%), menstrual complaints (11.3%), and pelvic inflammatory disease (38.4%). Of the antenatal women presenting to the OPD, almost 30% were primigravida and the majority were in their second trimester (55.8%).

### Impact of COVID-19

Approximately 33% of respondents faced difficulty in accessing health care in the form of lack of public transport (29%), financial difficulties (66.1%), or fear of being stopped by authorities during the lockdown. Of the 33% that experienced difficulty with access, 50% reported changes in their husbands’ behavior in the form of increased irritability due to difficulty procuring alcohol and tobacco during the lockdown. Sixty-five percent of the respondents reported a change in the family environment during the lockdown. Positive effects evidenced by increased family time and bonding were reported by 20% of respondents; the rest reported stressful impacts due to job loss, job insecurity, and other financial difficulties.

### Experience of Domestic Violence

Of the 412 women screened, 32.5% reported domestic violence in some form, with the majority being verbal abuse in the form of insults, threats of physical violence, or public humiliation (Table 2).

**TABLE 2.** Experience of Domestic Violence Among Women in 2 Health Care Facilities, Nagpur, India, June to August 2020

N=412	**Yes No. (%), n=134**	**More in the Last 3** **Months No. (%), n=134**
Only verbal	63 (47.0)	9 (6.7)
Only physical	17 (12.7)	3 (2.2)
Only sexual	8 (6.0)	1 (0.7)
Verbal and physical	22 (16.4)	2 (1.4)
Verbal, physical, and sexual	16 (12.0)	3 (2.2)
Verbal and sexual	8 (6.0)	2 (1.4)
Total	134 (32.5)	

**Verbal Abuse:** Verbal abuse was most commonly experienced, with 47.0% reporting only verbal abuse—mainly in the form of public humiliation and their partners insulting them or making them feel bad about themselves. Thirty-four percent of women reported verbal abuse along with physical or sexual abuse.

**FIGURE 1:**
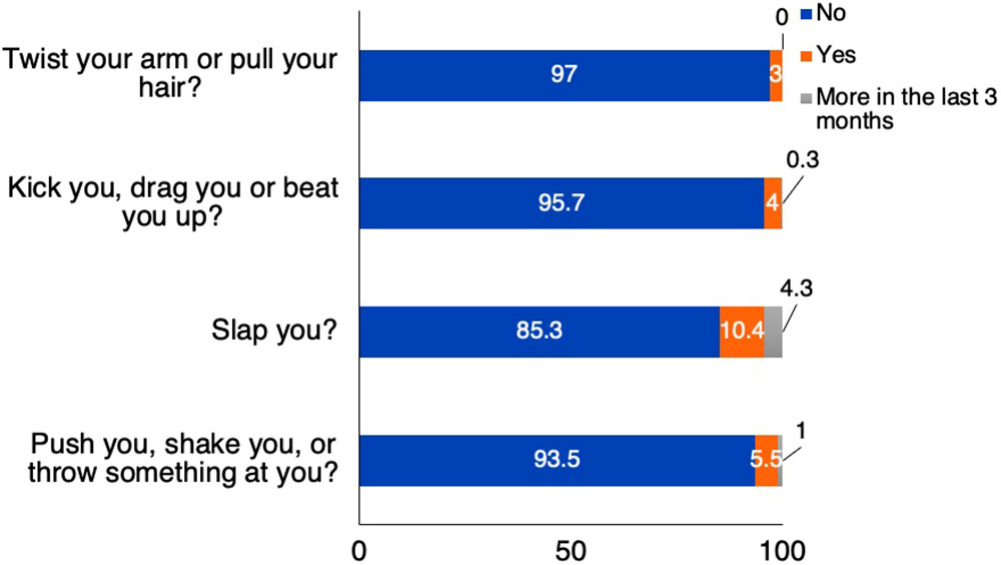
Details of Physical Abuse Among Women Who Experienced Domestic Violence, Nagpur, India

**Physical abuse** (Figure 1): The most common form of physical abuse was slapping, followed by pushing or shaking. One percent of women reported severe abuse in the form of serious burns, deep wounds, broken bones, broken teeth, or any other acute injury.

**Sexual abuse:** Eight women (6.0%) reported sexual abuse. The most common type of sexual abuse was her partner physically forcing the woman to have sexual intercourse even when she did not want to.

Of the 134 women who faced domestic violence, 15 were pregnant. The most common form of abuse reported during pregnancy was verbal abuse in the form of insults and humiliation by the husband. Of these, the majority (73.3%) were in their first trimester, and only 1 woman was in her third trimester.

The perpetrator in the majority of abuse cases was the woman’s husband (66.4%), followed by another family member such as her mother-in-law (25.4%), father-in-law (8.2%), and others, such as a sister-in-law (0.7%).

**FIGURE 2:**
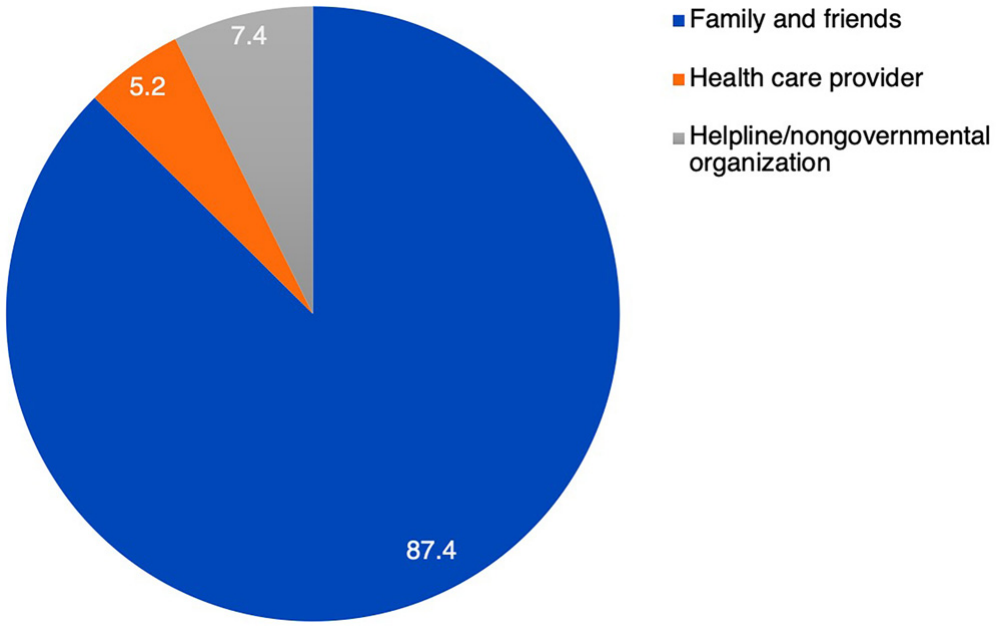
Types of Assistance Sought by Women Who Experienced Domestic Violence, Nagpur, India

It was noted that only 7.4% of women reported that they called helplines or lodged a formal complaint. Most (87.4%) reported that they confided in their family and friends (Figure 2).

### Inferential Analysis

The woman’s age and type of family were not significantly associated with history of domestic violence. However, education levels showed a significant association with violence during the pandemic. Participants with up to secondary education reported particularly high rates of domestic violence during the study. Women whose spouses indulged in substance abuse presented with higher rates of violence (Table 3).

**TABLE 3.** Inferential Analysis of Various Demographic Variables With Domestic Violence History Women in 2 Health Care Facilities, Nagpur, India, June to August 2020

	**Domestic Violence, No. (N=412)**		
	**Yes**	**No**	*P* Value
Mean age of woman, years	25±4.2	23±5.7	
Education			
Up to secondary school	8	41	
Higher education	48	237	<.001^a^
Type of family			
Nuclear	91	144	
Joint	43	134	.28
Husband’s addiction			
Yes	56	14	
No	78	264	<.001^a^

^a^Statistically significant.

## DISCUSSION

This study indicates a domestic violence prevalence of 32.5%, the majority of which was in the form of verbal abuse. This is higher than the findings in Maharashtra evidenced by NFHS-4 (2015–2016), which reported a prevalence of 21%.^2^ This is considerably higher than the findings by Sharma et al., who reported a prevalence of 8.5% in April 2020.^16^ This difference may be because Sharma et al. conducted an online survey in April, immediately after the imposition of the 2-week lockdown. A reason for the higher numbers reported in our study may be the nature of the interviews (in person), which allowed the investigators to enquire sensitively and in greater detail.

A study conducted among 250 pregnant women during the COVID-19 pandemic in Iran reported that 35.2% of the women were exposed to domestic violence, with the most common type being emotional violence.^5^ In contrast, in our study, 16.2% of the pregnant women attending the OPD were exposed to domestic violence. As per NFHS-4, 4% of women who have ever been pregnant have experienced physical violence during 1 or more pregnancies.^2^ In a study conducted in Nagpur among 600 pregnant women in 1999, the incidence of abuse among pregnant women was found to be 25.3%.^17^ This shows a reduction in the incidence of abuse among pregnant women in the region over 2 decades.

Musa et al. reported the prevalence of physical, emotional, and sexual violence as 25.9%, 25.6%, and 3.7%, respectively.^18^ Similarly, a community-based study conducted in Aurangabad also reported physical abuse as the most common form of domestic violence.^19^ In contrast, in our study, the most common type of violence was verbal abuse, followed by physical abuse, similar to Sharma et al.’s findings.^16^ Results reported by Hessami et al., Sarayloo et al., and Tavoli et al. were in line with our study, reporting higher levels of verbal violence and lower levels of physical violence.^20^^–^^22^

Multiple studies have established that social isolation and times of conflict increase the risk of victimization.^5^^,^^23^ There have been multiple reports of violence against women during the COVID-19 pandemic.^5^^,^^24^^,^^25^ Sediri et al. found higher scores of depression, anxiety, and stress among women who faced violence during the COVID-19 lockdown in Tunisia.^26^

The majority of women who experienced domestic violence sought help from family and friends, rather than the police or other institutions.

Most of the women sought help and solace from family members and friends while avoiding formal avenues such as helplines and nongovernmental organizations. This was confirmed by the findings of the NFHS-4, wherein only 9% of women who have ever experienced physical or sexual violence sought help; they also turned to friends and family.^2^ A study from Portugal also reported that 62% of women did not seek any help at all because they were embarrassed or felt that it would not make any difference.^27^ This finding was similar to a United Nations report stating that less than 40% of women victims of violence seek any sort of help, and primarily seek help from family or friends.^28^ This report also highlighted the fact that less than 10% of victims who request help do so from the police.^29^

Health care workers are overwhelmed by the clinical aspects of COVID-19 and managing the sheer burden of cases and deaths. However, they should be sensitized about the warning signs that may point toward domestic violence. Health care workers should ask community health workers—for example, accredited social health activists in India—to follow up on their beneficiaries as they live in the same community. Dedicated helpline numbers, email addresses, and WhatsApp numbers should be well advertised so that victims can report violence and seek help. In India, Fatke et al. reported clusters of patients presenting with psychiatric symptoms during COVID-19, including increased cases of domestic violence associated with increased drug or alcohol use in both victims and perpetrators.^29^

There is a need to engage nongovernmental organizations, civil societies, public health departments, psychologists, and allied mental health professionals to fill this gap. The Government needs to formally integrate domestic violence and mental health repercussions into public health preparedness and COVID-19 emergency response plans. The National Commission for Women has also reported an increased number of distress calls from women experiencing abuse.^11^ In Italy, there was a diametrically opposite phenomenon: calls to toll-free antiviolence numbers actually decreased. However, it is suspected that the decrease in reports did not reflect a reduction in violent incidents, but that, on the contrary, the greatest risk during the COVID-19 pandemic was that a victim remains trapped at home with her abuser, with no escape route or the opportunity to contact outside help.^16^

### Strengths and Limitations

The strength of the current study is its sample size. There have been few studies with such a large sample in the Indian subcontinent. Another advantage is the face-to-face nature of the interview, unlike the online surveys in many studies conducted during COVID-19. Since our study did not involve online surveys, we could overcome the limitations of digital literacy and Internet accessibility, thus targeting varied sectors of the population. One of the limitations is its cross-sectional nature and the format of hospital-based sampling; as a result, this may not represent the true scenario across communities. Community screening could not be done due to COVID-19 restrictions. Since it is a retrospective study, there are chances of recall and interviewer bias.

## CONCLUSION

Although the pandemic may be on the wane, the footprint of COVID-19 on psychological wellness and prolonged financial insecurity may perpetuate the same family environment, continuing the same exposure risk to the women. In conclusion, this study has demonstrated that, as has been proven time and again, there is an increase in domestic violence during various crises and conflict situations. Special attention needs to be given to this problem. As we prepare for the third wave of COVID-19 in India, including an ongoing partial lockdown and its repercussions on the financial and social fabric of society, there is a need to focus on screening women, generating community awareness, and creating an encouraging environment for women to come forward to report their abuse.

## Notes

**Peer Reviewed**

**First published online:** July 18, 2022.
